# Phytogenic Modulation of Rumen Fermentation Reshapes Amino Acid Metabolism in Weaned Dairy Calves: A Mechanistic Insight

**DOI:** 10.3390/ani16142265

**Published:** 2026-07-22

**Authors:** Heba M. Fouad, Alzahraa M. Abdelatty, Sobhy M. A. Sallam, Mahmoud M. El-Attrouny, Eman A. Elwakeel, Mohamed S. Yusuf, Yousef W. Fathy, Hossam A. Abdellatif

**Affiliations:** 1 Department of Nutrition and Clinical Nutrition, Faculty of Veterinary Medicine, King Salman International University, Ras Sadr 46612, Egypt; hebafouad3131@gmail.com (H.M.F.); yusufm282@vet.suez.edu.eg (M.S.Y.); 2 Department of Nutrition and Clinical Nutrition, Faculty of Veterinary Medicine, Cairo University, Giza 12211, Egypt; hossam12@hotmail.com; 3 Department of Animal and Poultry Production, College of Agriculture and Food, Qassim University, Buraydah 52571, Saudi Arabia; 4 Department of Animal Production, Faculty of Agriculture at Moshtohor, Benha University, Benha 13511, Egypt; mahmoud.elatrouny@fagr.bu.edu.eg; 5 Department of Animal and Fish Production, Faculty of Agriculture (El-Shatby), Alexandria University, Alexandria 21545, Egypt; emankeel@alexu.edu.eg; 6 Department of Nutrition and Clinical Nutrition, Faculty of Veterinary Medicine, Suez Canal University, Ismailia 41522, Egypt; 7 Green Milk Farm, Cairo Alexandria Dessert Road, Cairo 36000, Egypt; youssefwafeek110@gmail.com

**Keywords:** plant extract, growth performance, amino acid, metabolomics, molecular docking, dairy calves

## Abstract

The impact of essential oils on amino acid metabolism in relation to the rumen fermentation outcomes and adaptation to a solid diet is not well documented. Our objective was to evaluate the impact of an essential oil blend (EOB; contains cinnamaldehyde, capsicum, curcumin, and saponin) on growth performance, rumen fermentation, and serum amino acids of weaned growing calves. Sixteen weaned Holstein calves were blocked by body weight (100 ± 6 kg), and randomly allotted into: 1—CON group (*n* = 8; received 0% EOB), and 2—EOB group (*n* = 8; received 0.5 g of the EOB/Kg dry matter for 60 d, sex was used to balance the groups, so each group has 4 male and 4 female calves, but it is not the main scope of this study. Growth performance, rumen fermentation, and serum amino acids were assessed. Growth performance was not affected by treatment. Rumen pH decreased, and valeric acid, acetate, and propionate levels were increased. Serum leucine was reduced, and aspartate increased. Metabolic pathway enrichment analysis identified alanine, aspartate, and glutamate metabolism as the most affected metabolic pathway. In conclusion, supplementation with this EOB did not impact growth performance, despite its impact on rumen fermentation and blood amino acids in weaned Holstein calves. This study indicates that phytogenic supplementation may contribute to metabolic modulation during the post-weaning period through modulation of amino acid-associated metabolic pathways.

## Simple Summary

The post-weaning period is stressful for dairy calves due to the transition from a liquid (milk) to a solid-feed diet. This study demonstrates that supplementing an essential oil blend (EOB) of cinnamaldehyde, capsicum, curcumin, and saponin alters rumen fermentation and serum amino acids in weaned Holstein calves, which indicates that the EOB actively modifies microbial fermentation pathways. Notably, the reduction in serum leucine and increase in aspartate, coupled with enrichment of the alanine, aspartate, and glutamate metabolic pathways, suggest that phytogenic compounds might play a vital role in post-weaning metabolic modulation by modulating systemic nitrogen and amino acid utilization. These significant physiological and metabolic shifts were coupled with stable growth performance. This indicates that EOB supplementation at 0.5 g/kg of dry matter could be safely used as a functional feed additive to support calves during the critical post-weaning transition. These findings provide animal scientists and nutritionists with new insights into how essential oils could regulate amino acid metabolism. This research supports the development of targeted phytogenic strategies to optimize rumen development and metabolic resilience in growing calves.

## 1. Introduction

Weaning represents a highly critical and stressful transition phase in the life cycle of dairy calves, characterized by shifts from a combination of a liquid milk diet with/ without calf starter to completely solid feed post-weaning. This transition often triggers significant physiological challenges, including gastrointestinal microbial shift, fragile immune status, and temporary growth inconsistency [1].

During the pre-weaning period, phytogenic feed additives are widely recognized for their potent antimicrobial action, which effectively reduces the incidence and severity of diarrhea in young calves [2]. These phytogenic substances, which include essential oils, saponins, and oleoresins, have recently gained significant focus in ruminant production as sustainable, safe alternatives to conventional antibiotic growth promoters [3].

Beyond their antimicrobial properties, specific bioactive compounds such as cinnamaldehyde, capsicum, curcumin, and saponins have been shown to act as rumen modifiers. For instance, cinnamaldehyde can alter ruminal bacterial and archaeal populations to modify volatile fatty acid production [4]. Capsicum oleoresin modulates metabolic pathways and immune responses [5], while curcumin decreases the acetate-to-propionate ratio in the rumen fluid to enhance microbial fermentation efficiency [3].

Saponins are well documented to manipulate rumen fermentation patterns and improve nutrient utilization by suppressing ciliate protozoa [1]. When applied as a blend, these phytogenic compounds can exert synergistic benefits, improving overall gastrointestinal health, gut integrity, and growth performance [5,6]. Despite these established benefits of essential oils on rumen fermentation and changes in fermentation end products (Volatile fatty acids), how their impact on rumen fermentation will impact the amino acid profile after shifting to a solid diet during post-weaning is not well documented.

The diverse changes in rumen fermentation following essential oil supplementation were formerly reported to impact microbial protein metabolism [7], which could be reflected in the host protein and amino acid metabolism. A recent study reported an increase in some free amino acids, including leucine, isoleucine, lysine, valine, phenylalanine, tyrosine, alanine, glutamic acid, glycine, and cysteine, when *Allium mongolicum* essential oil was fed to finishing lambs without a noticeable impact on final body weight and feed intake [8], which indicates the role of essential oils in regulating host protein metabolism.

A deeper understanding of these metabolic interrelationships is essential to optimize dietary strategies that support the metabolic modulation of growing weaned calves. Therefore, the objective of the current study was to evaluate the impact of a specific phytogenic blend containing cinnamaldehyde, capsicum, curcumin, and saponin on the growth performance, rumen fermentation characteristics, and serum amino acid profiles of weaned growing Holstein calves.

We hypothesized that dietary supplementation with this essential oil blend (EOB) would favorably modulate ruminal fermentation parameters and alter nitrogen-associated metabolic pathways, thereby providing a physiological framework for enhanced metabolic modulation during the critical post-weaning transition.

## 2. Materials and Methods

### 2.1. Animal Welfare Statement

The authors confirm that all the experimental procedures were approved by the Institutional Animal Care and Use Committee of the Faculty of Veterinary Medicine, Cairo University, on 13 October 2024. (Approval number vet CU13102024985), The experiment was conducted at a private dairy farm (Green milk Dairy farm) in Egypt.

### 2.2. Animals, Experimental Design, and Treatments

For a 60 d experimental period, sixteen Holstein weaned calves were blocked by their body weight, and randomly distributed into two treatment groups: 1—CON (*n* = 8); 0 g of EOB, and 2—EOB (*n* = 8); 0.5 g of the EOB/Kg dry matter (top-dressing; on top of the diet). The calves were 8 males and 8 females; sex was used to balance the calves between groups, and was not the main target blocking criterion. Therefore, each group had 4 males and 4 females, and each block had the same sex in each group. The phytogenic product Actifor Boost^®^ (Delacon Biotechnik GmbH; Steyregg, Austria) contains Cayenne pepper extract (Capsaicin; 100 mg), Curcuma extract (Curcumin; 1000 mg), Cinnamon extract (Cinnamaldehyde; 2000 mg), and Quillaja extract (Saponin; 3000 mg). The carrier materials are wheat bran (250 g) and limestone (330 g). The dose of the product was selected based on the manufacturer's instructions.

The basal diet was formulated to meet the nutrient requirements of growing Holstein Calves; the diet ingredients and chemical composition are shown in Supplementary Table S1. Calves were housed in individual pens with water provided all the time, and feed was provided twice daily.

### 2.3. Growth Performance

The feed offers and refusals were measured daily to calculate the daily dry matter intake. Body weight was recorded weekly. Daily body weight gain was calculated by subtracting the initial body weight from the final body weight and dividing by the experimental period (60 d). Feed efficiency was then calculated by dividing the kg of BW gain/kg of dry matter intake, similar to Mandouh et al. [9].

### 2.4. Rumen pH and Volatile Fatty Acids

At the end of the experiment, rumen fluid was collected through an oral stomach tube (Ancitech, Winnipeg, MB, Canada) 4 h after morning feeding, similar to [10]. The initial 30 mL of rumen fluid was discarded to avoid saliva contamination, and then rumen pH was immediately measured using portable pH meter (pH600, Adwa, Szeged, Hungary), rumen fluid then was filtered via four layers of cheesecloth and then 0.2 mL of 25% metaphosphoric acid was added to 1.0 mL of rumen fluid samples and stored at −20 °C for volatile fatty acid (VFA) analysis. The VFA contents then were measured using gas chromatography (6890 N; Agilent Technologies, Avondale, PA, USA), similar to [11].

### 2.5. Blood Sampling

Blood samples were collected at the end of the experiment, before morning feeding, via jugular venipuncture, similar to [12]; serum aliquots were stored at −20 °C till analysis.

### 2.6. Serum Amino Acids Assessment

The free amino acids were quantified using high-performance liquid chromatography (HPLC; Agilent 1200 HPLC System, Agilent Technologie, Santa Clara, CA, USA). Phenylisothiocyanate (PITC) pre-column derivatization, as previously described [13]. Samples were homogenized in 75% aqueous methanol (1:10 *w*/*v*), followed by centrifugation at 4000 rpm for 10 min. The clear supernatant was divided for analysis and subjected to evaporation under vacuum. The derivatization mixture was prepared from methanol, TEA, distilled water, and PITC at a 7:1:1:1 ratio and added to the sample.

Chromatographic separation was performed using an Agilent 1200 HPLC system equipped with a quaternary pump, UV detector, and PICO-TAG C18 column (3.9 × 30 cm). The mobile phase included eluent buffers provided in the Waters amino acid chemistry kit (Water Corporation, Milford, MA, USA). Elution was carried out at 46 °C with a flow rate of 2.0 mL/min and detection at 250 nm. Each derivatized sample (20 μL) was injected, and amino acids were identified and quantified by comparison with known standards processed similarly.

### 2.7. Metabolic Pathway Enrichment Analysis

Metabolic pathway enrichment analysis was performed using MetaboAnalyst 6.0 (Xia Lab, McGill University, Montreal, QC, Canada). Differential serum metabolites identified between the control and phytogenic-supplemented calves were subjected to pathway analysis. Metabolite identities were standardized and matched against the Human Metabolome Database (HMDB) and the Kyoto Encyclopedia of Genes and Genomes (KEGG) libraries integrated within MetaboAnalyst.

Pathway analysis was conducted using the Pathway Analysis module with Bos taurus selected as the reference organism. Enrichment analysis was performed using the Global Test algorithm, while pathway topology analysis employed relative-betweenness centrality to estimate pathway impact. Pathways were ranked according to enrichment significance (*p*-value) and pathway impact values.

KEGG-based metabolic pathway maps, enrichment scatter plots, and pathway interaction networks were generated within MetaboAnalyst to visualize biologically relevant metabolic alterations associated with phytogenic supplementation. Particular emphasis was placed on pathways associated with amino acid metabolism and nitrogen metabolism due to their biological relevance to ruminal fermentation and host metabolic modulation.

### 2.8. Molecular Docking Analysis

Molecular docking analysis was conducted to investigate the potential interaction between quillaic acid, the principal triterpenoid aglycone of Quillaja saponins, and glutamate dehydrogenase (GDH). GDH was selected because pathway enrichment analysis identified alanine, aspartate, and glutamate metabolism as the most significantly affected pathway, and GDH is a central enzyme linking glutamate metabolism with nitrogen metabolism. Quillaic acid was chosen as the representative ligand because it is the major bioactive aglycone of Quillaja saponins, one of the principal active constituents of the phytogenic blend. The three-dimensional structure of quillaic acid (CID: 10114) was retrieved from PubChem [14]. The ligand structure was protonated, energy-minimized using the MMFF94 force field, and converted into PDBQT format using Open Babel v3.1.1 [15].

The crystal structure of glutamate dehydrogenase (Clostridium symbiosum) was obtained from the RCSB Protein Data Bank (1BGV) [16]. Protein preparation was carried out using UCSF Chimera v1.17.3 [17], with additional structural inspection performed in PyMOL v2.5 and BIOVIA Discovery Studio Visualizer 2021. Water molecules, salts, and non-essential heteroatoms were removed, polar hydrogen atoms were added, and the prepared receptor structure was saved in PDB format before conversion to PDBQT format in PyRx v0.8, integrating AutoDock Vina v1.1.2 [18].

Docking simulations were performed using a rigid-receptor and flexible-ligand protocol in AutoDock Vina. A blind docking strategy was applied to avoid bias toward predefined binding pockets and to allow comprehensive exploration of potential ligand-binding regions across the entire GDH structure. The docking grid box was adjusted to cover the whole protein with the following coordinates and dimensions: center X = −16.7454, Y = 22.6498, Z = 20.0206; dimensions X = 63.1429 Å, Y = 52.5414 Å, and Z = 62.7260 Å. Docking calculations were performed using the default AutoDock Vina parameters with an exhaustiveness value of eight, and nine output poses were generated for each run.

The best docking poses were selected based on binding affinity, interaction quality, steric feasibility, and localization within catalytically relevant regions of the enzyme. Ligand–protein interactions, including hydrogen bonds, hydrophobic interactions, van der Waals contacts, and carbon–hydrogen bonds, were analyzed and visualized using PyMOL v2.5 and BIOVIA Discovery Studio Visualizer 2021.

### 2.9. Statistical Analysis

Statistical power analysis was performed for ADG as one of the most reliable parameters to consider in calf performance. Based on prior studies [4,19], the power was equal to 80%, and the α-level was 0.05. The minimal sample size to detect differences under this assumption was computed to be 8 calves per treatment group.

Normal distribution of data was tested using the UNIVARIATE procedure of SAS 9.4 (SAS Institute Inc., Cary, NC, USA). Mixed procedures (PROC MIX) with treatment and sex as the fixed effects and calf block as the random effect using the following model:

*Y_ijk_* = *µ* + *B_i_* + *T_j_* + *S_k_* + (*T* × *S*)*_jk_* + *ε_ijk_*

where *Y_ij_* represents the response variable of interest, *μ* is the overall mean, *B_i_* is the random effect of block, *T_j_* is the fixed effect of treatment, *S_k_* is the fixed effect of sex, *(T* × *S)_jk_* is the fixed interaction between sex and treatment, and *ε_ijk_* is the residual error.

The treatment means were compared using the Tukey–Kramer adjustment test within each family when making pairwise comparisons of treatments. Statistical differences were declared at *p* ≤ 0.05, and a tendency toward significance was considered at 0.05 < *p* ≤ 0.10.

## 3. Results

### 3.1. Growth Performance

The graphical illustration of all the results is shown in the graphical abstract. Under the current experimental condition, the phytogenic blend at the dose of 0.5 g/Kg dry matter did not have a statistically detectable difference on growth performance indices of the weaned calves (*p* > 0.05; Table 1). There was no impact of sex or sex by treatment interaction on any of the measured parameters (*p*-value for sex and treatment by sex was >0.15 for all the measurements).

### 3.2. Rumen Fermentation

**Figure 1:**
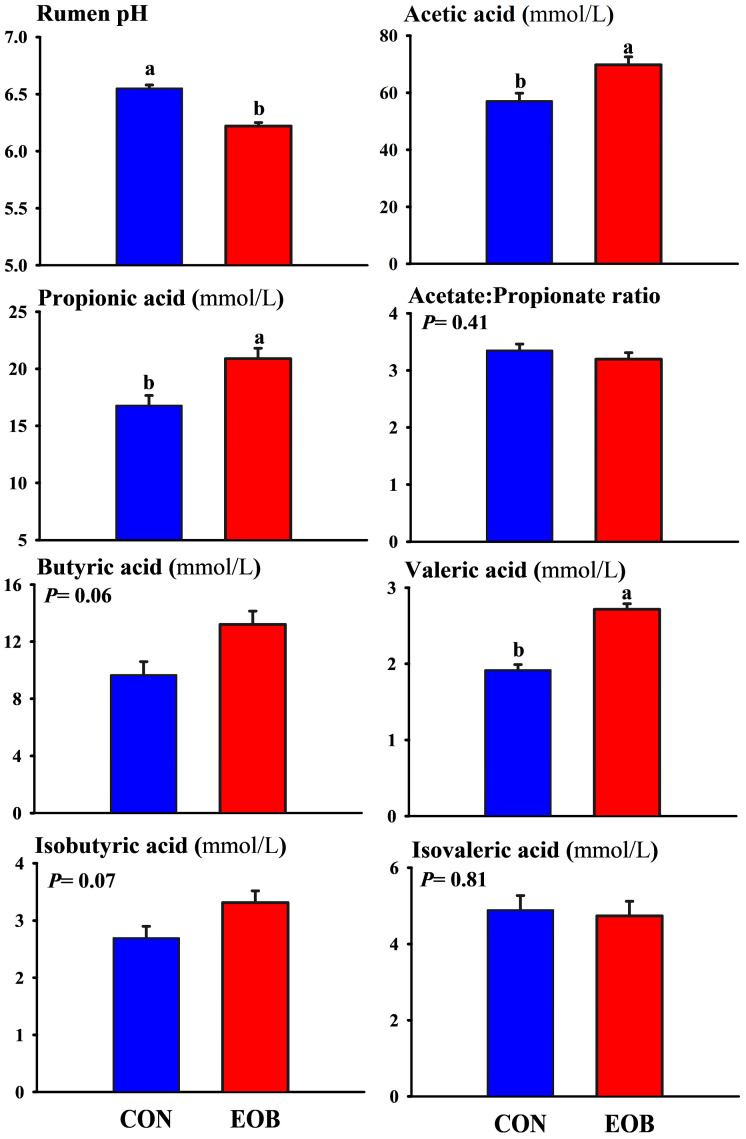
The effect of a phytogenic blend containing capsicum, curcumin, cinnamaldehyde, and saponin on rumen pH and volatile fatty acid production in weaned growing dairy calves. The rumen pH decreased (*p* = 0.01) in the group fed on a diet supplemented with the phytogenic blend (EOB) compared to the control group that received the basal diet (CON). The level of acetate, propionate, and valerate increased (*p* < 0.05), while butyrate and iso-butyrate levels tended to increase (*p* = 0.06 and 0.07, respectively); the *p*-value for sex and treatment by sex was >0.20 for all the rumen fermentation parameters. Different letters indicate significant *p*-value.

Supplementation of the weaned dairy calves’ diet with EOB affected rumen fermentation indices (Figure 1). Rumen pH was within the normal range for all groups [20], and it was decreased (*p* = 0.01) by ~0.3 units in the EOB group (pH 6.2) compared to the CON group (pH 6.5). In addition, the level of acetic and propionic acids increased in the EOB group (by 22.3% and 24.4%, respectively) relative to the CON counterpart (*p* < 0.05); however, the acetate-to-propionate ratio was not statistically different between groups (*p* > 0.1). The level of valeric acid was higher (*p* < 0.01) in the EOB group, while both butyric and isobutyric acid tended to increase (*p* = 0.06 and 0.07, respectively) in the EOB group compared to CON calves.

### 3.3. Serum Amino Acid Profile

Serum amino acid profile response to phytogenic feed additive supplementation is presented in Table 2. The level of Aspartic acid increased (*p* < 0.05), while Lysine and Tryptophan tended to increase in the EOB group (*p* = 0.09 and 0.08, respectively). On the other side, the level of Leucine decreased by 14% in the EOB group compared to the CON counterpart (*p* = 0.02).

### 3.4. Metabolic Pathway Enrichment Analysis

**Figure 2:**
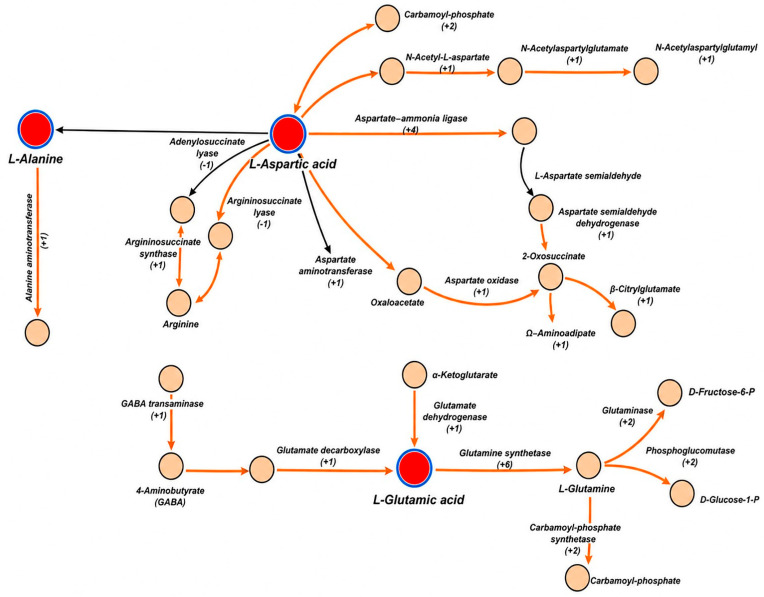
KEGG-based pathway map of alanine, aspartate, and glutamate metabolism generated using MetaboAnalyst 6.0 with the *Bos taurus* pathway library. Differential serum amino acids identified between the control and phytogenic-supplemented calves were mapped onto the corresponding KEGG pathway. Red-highlighted nodes represent metabolites detected in the present study (L-alanine, L-aspartic acid, and L-glutamic acid). Only metabolites belonging to this significantly enriched pathway are displayed by the KEGG pathway map; therefore, not all quantified amino acids are shown.

Metabolic pathway enrichment analysis revealed significant modulation of amino acid-associated metabolic pathways in calves supplemented with the phytogenic blend (Figure 2; Table 3). KEGG-based pathway enrichment analysis performed in MetaboAnalyst identified alanine, aspartate, and glutamate metabolism as the most significantly enriched pathway (*p* = 0.04; pathway impact = 0.42). This pathway was primarily driven by alterations in serum aspartic acid, alanine, and glutamic acid concentrations.

Additional enriched pathways included arginine biosynthesis (*p* < 0.01), histidine metabolism (*p* < 0.01), and nitrogen metabolism (*p* = 0.03). Pathways associated with branched-chain amino acid metabolism, including valine, leucine, and isoleucine biosynthesis, were also affected by phytogenic supplementation.

The KEGG pathway mapping indicated coordinated modulation of amino acid interconversion and nitrogen-associated metabolic processes, suggesting that phytogenic supplementation may influence intermediary metabolic pathways related to ruminal fermentation and host metabolic modulation in weaned dairy calves.

### 3.5. Molecular Docking

**Figure 3:**
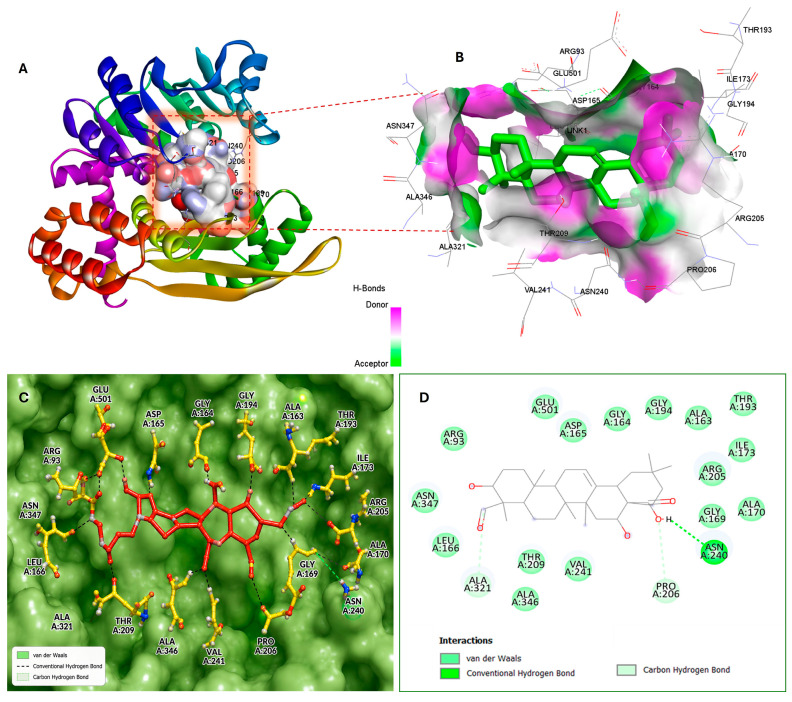
Post-weaning supplementation of essential oil blend for dairy calves. Molecular docking interaction of quillaic acid (Quillaja Saponin) with glutamate dehydrogenase (**A**). Overall three-dimensional structure of the GDH–quillaic acid complex. (**B**) Surface representation of the binding pocket showing ligand accommodation within the active site. (**C**) Three-dimensional interaction map illustrating hydrogen bonding and hydrophobic interactions between quillaic acid and key amino acid residues of GDH, where carbon atoms are shown in yellow, oxygen atoms are shown in red, and nitrogen atoms are in blue. (**D**) Two-dimensional schematic diagram of ligand–protein interactions, including conventional hydrogen bonds, carbon–hydrogen bonds, and van der Waals interactions.

Molecular docking analysis revealed a strong interaction between quillaic acid and glutamate dehydrogenase, with a binding affinity of −9.2 kcal/mol. The overall three-dimensional structure of the docked complex demonstrated the localization of quillaic acid within the GDH binding cavity (Figure 3A). Surface representation analysis further confirmed stable accommodation of the ligand inside the interaction pocket and showed the distribution of hydrogen bond donor and acceptor regions surrounding the ligand (Figure 3B).

Detailed interaction analysis indicated that quillaic acid formed a conventional hydrogen bond with Asn240, in addition to carbon–hydrogen bond interactions involving Leu166 and Pro206. Several additional residues contributed van der Waals and hydrophobic interactions, including Arg93, Glu501, Asp165, Gly164, Gly194, Ala163, Thr193, Ile173, Ala170, Arg205, Gly169, Thr209, Ala321, Ala346, Val241, and Asn347 (Figure 3C).

The two-dimensional interaction map summarized the conventional hydrogen bonds, carbon–hydrogen bonds, and van der Waals contacts formed between quillaic acid and the amino acid residues within the GDH binding pocket (Figure 3D).

## 4. Discussion

In the current study, the EOB was fed for 60 d experimental period, which changed the level of acetate and propionate, but the overall rumen acetate:propionate ratio did not change, indicating their selective antimicrobial action inside the rumen. This might explain why EOB did not have a statistically significant effect on growth performance in the current study [21], for instance, capsicum has selective antimicrobial action against both Gram-positive and Gram-negative bacteria [21], unlike ionophores, which have a very well-established distinctive effect against Gram-positive bacteria, and reduce both energy and protein losses, thus preserving those nutrients for body development [21].

Concurrently, the noticeable changes in some serum amino acids are in line with changes in branched chain volatile fatty acids, indicating a selective role of the used EOB on rumen bacteria regulating protein degradation and the amino acids deamination process [7]. The integration of rumen fermentation profiling, serum amino acid profile analysis, metabolic pathway enrichment, and molecular docking in the current study provides a broader mechanistic perspective regarding how phytogenic compounds may influence nitrogen and amino acid metabolism during the post-weaning adaptation period in dairy calves. Because the phytogenic product consisted of multiple bioactive compounds, the present study cannot attribute the observed metabolic responses to any single constituent. Instead, the observed effects likely reflect the combined or synergistic actions of cinnamaldehyde, capsicum, curcumin, and Quillaja saponins.

Although phytogenic supplementation has altered rumen fermentation and amino acid metabolism, these changes were not accompanied by measurable improvements in growth performance during the study period. A similar observation was previously reported in healthy calves, where phytogenic supplementation altered ruminal and metabolic parameters without significantly affecting body weight gain [22].

The pathway enrichment analysis further supported the hypothesis that altered ruminal fermentation was associated with coordinated modulation of intermediary metabolic pathways, particularly alanine, aspartate, and glutamate metabolism. These pathways are closely associated with carbon flux, ammonia assimilation, and amino acid interconversion, suggesting a potential link between ruminal fermentation products and nitrogen metabolism. To our knowledge, limited information is available regarding the effects of phytogenic blends on serum amino acid profile in weaned dairy calves; an in-depth study is still required to unravel the molecular mechanism driving their role in protein metabolism.

Deamination of amino acids in the rumen results in the production of ammonia and VFA [23]. As formerly reported, essential oils play a role in rumen protein metabolism by either decreasing the amino acid deamination rate [7] or decreasing the rate of protein breakdown [24,25]. For instance, cinnamaldehyde was reported to inhibit amino acid deamination [26], thus reducing the level of branched chain VFA and increasing the level of amino acids escaping the rumen, which could be reflected in the serum amino acid profile. The same findings were reported with curcumin [27] and Saponin [6]. The increased serum aspartate concentration together with altered leucine, lysine, and tryptophan levels observed in the present study may therefore indicate modifications in amino acid turnover and nitrogen utilization associated with phytogenic-induced shifts in ruminal metabolism. Moreover, the enrichment of alanine, aspartate, and glutamate metabolism identified in the present study suggests that phytogenic supplementation may influence metabolic pathways involved in amino acid transamination and glutamate-associated nitrogen cycling.

In another report, a blend of cinnamaldehyde and eugenol increased the level of isobutyrate, indicating some activity on amino acid deamination [7]. On the same line, capsicum increase the level of rumen valeric and butyric volatile fatty acids [28], indicating its ability to modulate amino acid deamination (microbial breakdown of amino acids) in the rumen, thus producing more valerate and butyrate, which is in line with our findings; however, under the current experimental design, we observed only tendency to increase in butyric and isobutyric acids. Valerate and branched-chain volatile fatty acids are considered indicators of amino acid fermentation and branched-chain amino acid metabolism in the rumen [23]. Therefore, the observed increase in valerate together with altered leucine metabolism may further support the involvement of phytogenic compounds in regulating ruminal amino acid degradation pathways.

Additionally, when a higher level of rumen unprotected leucine was added to the ration of Xiangxi yellow cattle, there was an increase in the production of valerate and isobutyrate [29], and it increased serum leucine level above that of the control group; this finding could explain the decrease in serum leucine level noted in the current study [30]. Interestingly, pathway enrichment analysis also identified valine, leucine, and isoleucine biosynthesis as an affected pathway, suggesting that branched-chain amino acid metabolism may represent an additional metabolic target of phytogenic supplementation.

In addition to its role in changing rumen environment, essential oils were found to play a role in amino acid transporter genes in the intestine; for instance, capsaicin improved serum amino acid profile by increasing the abundance of the amino-acid transporter genes in the intestine of pigs [31]. Since lysine is a basic amino acid, tryptophan is an aromatic amino acid, and aspartate is an acidic amino acid [32], each has a specific intestinal transporter mechanism, indicating the possible ability of phytogenic supplements to act on different intestinal protein transporters [33]. Furthermore, molecular docking analysis demonstrated a strong interaction between quillaic acid and glutamate dehydrogenase (GDH), a key enzyme involved in glutamate metabolism and ammonia assimilation. GDH plays a central role in linking carbon and nitrogen metabolism through reversible conversion between glutamate and α-ketoglutarate. The observed binding affinity between quillaic acid and GDH may therefore provide mechanistic support for the pathway enrichment findings related to glutamate-associated metabolic pathways and nitrogen metabolism. Although docking analysis alone cannot confirm direct biological inhibition, these findings collectively suggest that phytogenic compounds may influence amino acid-associated metabolic regulation through modulation of glutamate-centered metabolic processes. Studies incorporating rumen microbiome and metabolomic analyses are warranted to further elucidate the molecular mechanisms underlying phytogenic-mediated metabolic modulation in weaned dairy calves.

## 5. Conclusions

In conclusion, phytogenic supplementation modulated rumen fermentation and affected serum amino acid profiles in weaned dairy calves without affecting growth performance. Pathway enrichment analysis identified alanine, aspartate, and glutamate metabolism as the major affected pathway, suggesting modulation of nitrogen and amino acid metabolism. Molecular docking further supported a potential interaction between quillaic acid and glutamate dehydrogenase, providing mechanistic insight into phytogenic-associated metabolic regulation during post-weaning adaptation.

## Figures and Tables

**Table 1:** The impact of phytogenic blend on growth performance indices and body morphometry of weaned dairy calves ^1^.

Item	CON ^2^	EOB ^3^	SEM	*p*-Value *
Body weight, Kg	160	156	4.14	0.85
Body weight gain, Kg/d	0.99	0.90	0.05	0.32
Dry matter intake, Kg/d	4.15	4.21	0.20	0.84
Feed efficiency	0.25	0.22	0.02	0.25

^1^ Values are least square means; the phytogenic blend contains capsicum, cinnamaldehyde, curcumin, and saponins. ^2^ Control group received basal diet (*n* = 8). ^3^ Group received basal diet supplemented with phytogenic product (Actifor Boost^®^, *n* = 8). * *p*-value for treatment effect was considered significant at *p* ≤ 0.05. *p*-value for sex and treatment by sex was >0.20 for all the measurements.

**Table 2:** The impact of phytogenic blend on serum amino acid profile of weaned dairy calves ^1^.

Amino Acid (µg/mL)	CON ^2^	EOB ^3^	SEM	*p*-Value *
Alanine	15.2	12.03	1.64	0.31
Arginine	14.6	14.3	1.11	0.84
Aspartic acid	16.1	23.7	2.98	0.05
Cystine	2.02	2.01	0.20	0.94
Glutamic acid	32	41.8	4.06	0.22
Glycine	15.4	14	1.56	0.61
Histidine	5.41	5.5	0.14	0.47
Isoleucine	5.47	6.85	0.63	0.26
Leucine	5.76	4.93	0.13	0.02
Lysine	20	22.1	0.51	0.09
Methionine	4.1	4.14	0.30	0.95
Phenylalanine	11	10.5	2.18	0.86
Proline	12.3	12.2	0.89	0.95
Serine	8	9.93	0.81	0.22
Threonine	5.5	7.38	1.07	0.34
Tyrosine	7.42	7.53	0.47	0.88
Tryptophan	1.89	2.68	0.20	0.08
Valine	10.4	10.3	0.81	0.97

^1^ Values are least square means, phytogenic blend contains capsicum, cinnamaldehyde, curcumin, and saponins. ^2^ Control group received basal diet (*n* = 8). ^3^ Group received basal diet supplemented with phytogenic product (Actifor Boost^®^, *n* = 8). * *p*-value for treatment effect was considered significant at *p* ≤ 0.05. *p*-value for sex and treatment by sex was >0.15 for all the amino acids.

**Table 3:** Major metabolic pathways altered by phytogenic supplementation in weaned dairy calves based on MetaboAnalyst pathway enrichment analysis.

Pathway	Hits/Total	*p*-Value
Alanine, aspartate, and glutamate metabolism	3/28	0.04
Arginine biosynthesis	2/14	<0.01
Histidine metabolism	2/16	<0.01
Nitrogen metabolism	1/6	0.03
Valine, leucine, and isoleucine biosynthesis	1/8	<0.05

## Data Availability

The original contributions presented in this study are included in the article/supplementary material. Further inquiries can be directed to the corresponding authors.

## Supplementary Materials

The following supporting information can be downloaded at: https://www.mdpi.com/article/10.3390/ani16142265/s1, Table S1: Ingredients and chemical composition of the basal diet (% of DM, unless otherwise stated).
