# Supplementary material for: Phytogenic Modulation of Rumen Fermentation Reshapes Amino Acid Metabolism in Weaned Dairy Calves: A Mechanistic Insight
Source: Animals (Basel). 2026 Jul 22;16(14):2265. doi: 10.3390/ani16142265 (PMC13405558; doi:10.3390/ani16142265)
Supplement: Supplementary file 1 [file animals-16-02265-s001.zip › animals-4392827-supplementary.pdf]

Type of the Paper (Article)

Phytogenic modulation of rumen fermentation reshapes amino acid metabolism in weaned dairy calves: metabolomics pathway and molecular docking

Heba M. Fouad[1], Alzahraa M. Abdelatty[2*], Sobhy M. A. Sallam[3*], Mahmoud M. El-Attrouny[4], Eman A. Elwakeel[5], Mohamed S. Yusuf[1,6], Yousef W. Fathy[7], Hossam A. Abdellatif[2]

[1]Department of Nutrition and Clinical Nutrition, Faculty of Veterinary Medicine, King Salman International University, Ras-Sadr, South Sinai, Egypt. Hebafouad3131@gmail.com
[2]Department of Nutrition and Clinical Nutrition, Faculty of Veterinary Medicine, Cairo University, Giza 12211, Egypt; alzahraa@cu.edu.eg; raramehmet@gmail.com
[3]Department of Animal and Poultry production, College of Agriculture and Food, Qassim University, Saudi Arabia; so.sallam@qu.edu.sa
[4]Department of Animal Production, Faculty of Agriculture at Moshtohor, Benha University, Qalyubia, Egypt. mahmoud.elatrouny@fagr.bu.edu.eg
[5]Department of Animal and Fish Production, Faculty of Agriculture (El-Shatby), Alexandria University, Alexandria 21545, Egypt. emankeel@alexu.edu.eg
[6]Department of Nutrition and Clinical Nutrition, Faculty of Veterinary Medicine, Suez Canal University, Ismailia, Egypt. yusufm82@vet.suez.edu.eg
[7]Green Milk Farm, Cairo Alexandria dessert road, Cairo, Egypt. youssefwafeek10@gmail.com

Corresponding author: Alzahraa M. Abdelatty.
Corresponding author email: AMA: alzahraa@cu.edu ; raramehmet@gmail.com. SMAS: so.sallam@qu.edu.sa

Supplementary material

Supplementary Table S1

Table S1. Ingredients and chemical composition of the basal diet (% of DM, unless otherwise stated)

Ingredient	% of DM[1]
Alfalfa hay	37.9
Ground corn	26.5
Corn gluten feed	4.8
Soybean meal (46% CP)	16.7
Soybean hulls	12.9
Sodium bicarbonate	0.82
Vitamin-mineral Premix	0.30
Salt	0.08
Chemical composition	
DM (as-fed basis)	89.20
CP[2]	16.63

ME, Mcal/kg[3]	2.45
NDF[4]	31.42
Starch	18.31
Total fatty acids	2.21
Calcium	0.79
Phosphorus	0.35

[1]Dry matter

[2]Crude protein

[3] Metabolizable energy

[4]Neutral detergent fiber
